# Supplementary material for: Deconvoluting Cr states in Cr-doped UO2 nuclear fuels via bulk and single crystal spectroscopic studies
Source: Nat Commun. 2023 Apr 28;14:2455. doi: 10.1038/s41467-023-38109-0 (PMC10147638; doi:10.1038/s41467-023-38109-0)
Supplement: Supplementary file 1 — Supplementary Information file [file 41467_2023_38109_MOESM1_ESM.pdf]

Supporting Information

Deconvoluting Cr States in Cr-Doped UO_2 Nuclear Fuels via Bulk and Single Crystal Spectroscopic Studies

Murphy et al.

Corresponding authors:

Gabriel L. Murphy (g.murphy@fz-juelich.de)

Nina Huittinen (n.huittinen@hzdr.de)

Supplementary Information Note 1. Synchrotron X-ray Powder Diffraction

Supplementary Figure 1 displays Rietveld refinement profiles for Cr doped UO_2 (0 and 3500 ppm Cr_2O_3 addition) made against synchrotron X-ray diffraction (SXRD) data at ambient temperature obtained at BM20 ROBL ESRF. Measured samples are single phase fluorite structures in space group $Fm\bar{3}m$.

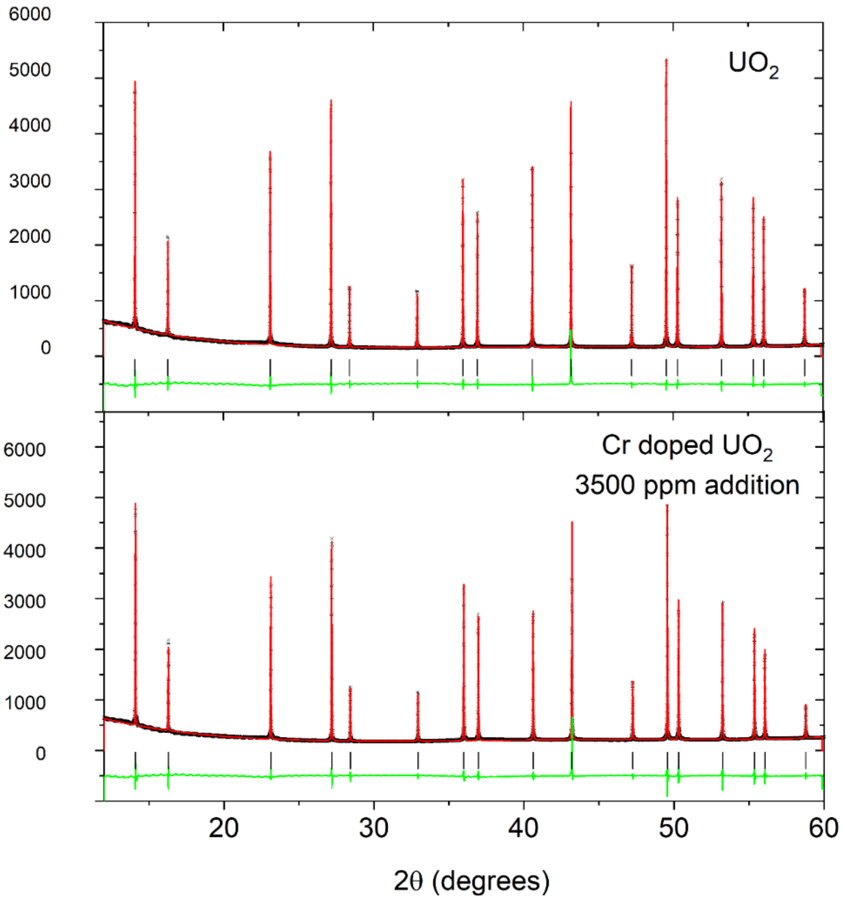

Supplementary Figure 1. Rietveld fit of the SXRD data at ambient temperature for UO_2 with 0 (top) and 3500 (bottom) ppm Cr_2O_3 addition. The black crosses, upper red line, lower green line and vertical black markers respectively represent observed data, calculated profile, difference profile and allowed reflections for UO_2 according to the space group $Fm\bar{3}m$. For UO_2 and Cr doped UO_2 with 3500 ppm addition the refined unit cell volumes are 163.2272(3) and 163.1647(5) Å respectively, where the respective wR_p = 5.108 and 5.850 % for λ = 0.7740 Å.

Supplementary Information Note 2. Scanning Electron Microscopy and Energy Dispersive Spectroscopy

Supplementary Figure 2 displays back scattered electron (BSE) microscope images collected on polished surfaces of Cr doped UO_2 pellets. Supplementary Table 1 provides grain size analysis of UO_2 pellets doped with 0 and 3500 ppm Cr_2O_3.

Supplementary Table 1. Minimum, maximum, mean grain sizes, and number of analyzed grains of the UO_2 pellets doped with 0 ppm and 3500 ppm Cr_2O_3.

	0 ppm	3500 ppm
Minimum [µm]	0.4	4.9
Maximum [µm]	41.7	290.1
Mean [µm]	5.0	103.4
Number of grains	1745	401

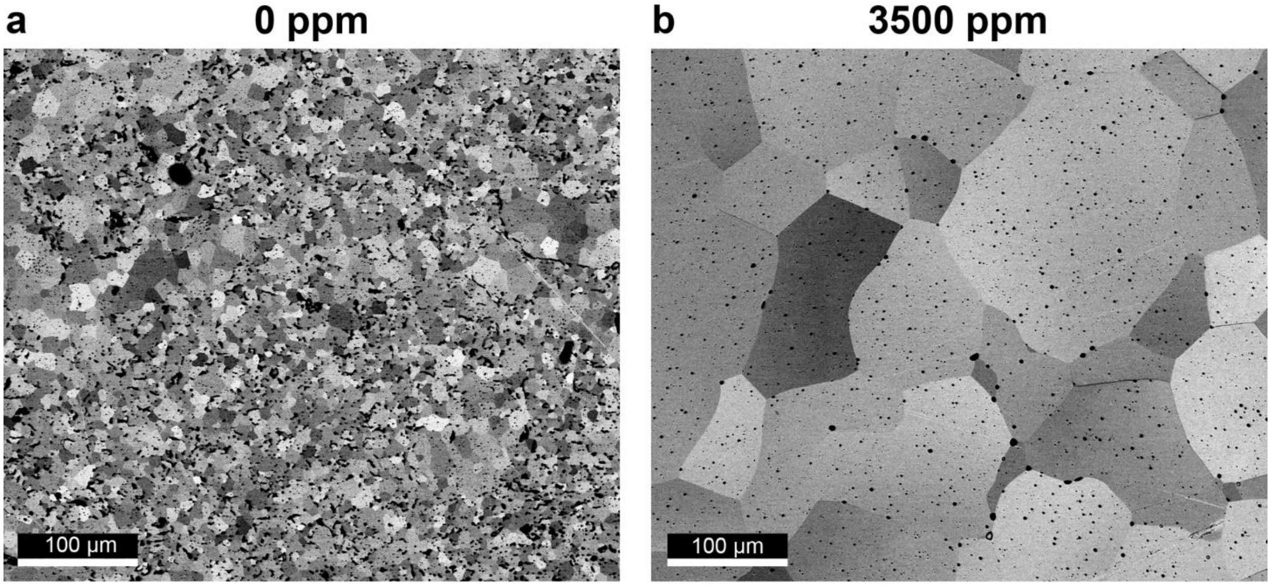

Supplementary Figure 2. BSE images of Cr doped UO_2 pellets (a) 0 ppm and (b) 3500 ppm Cr_2O_3 addition sintered at 1700 °C using an oxygen potential of -420 kJ/mol O_2.

Supplementary Information Note 3. Single Crystal X-ray Diffraction

Supplementary Table 2 provides the structural solution for the Cr doped UO_2 single crystal (SC1) shown in Figure 1 of the manuscript and also measured via EPR, HERFD-XANES and EXAFS (Figure 2a,b and d), indicating the occurrence of the Cr doped UO_2 fluorite structure in space group $Fm\bar{3}m$. No attempts were made to refine the Cr position. Supplementary Table 3 provides the structural solution for a Cr doped UO_2 single crystal (SC2) also examined via HERFD-XANES but found to contain Cr^o impurities. The refinement returned larger R factors for SC2 compared to SC1.

Supplementary Table 2. Crystallographic data for a Cr doped UO_2 single crystal grain.

**Compound**	Cr-doped UO_2
Formula	UO_2
Formula weight (g mol^{-1})	270.03
Crystal system	Cubic
Space group	$Fm\bar{3}m$
a (Å)	5.4410(3)
b (Å)	5.4410(3)
c (Å)	5.4410(3)
α (°)	90
β (°)	90
γ (°)	90
Volume (Å^3)	161.08(3)
Z / μ (mm^{-1})	4
F(000)	432.0
d_{calcd} (g cm^{-3})	11.135
GOF	1.063
Final R_1 [a] [$I > 2\sigma(I)$]	0.0193
Final wR_2 [b] [$I > 2\sigma(I)$]	0.0380

[a] $R_1 = \sum||F_o| - |F_c||/\sum|F_o|$,

[b] $wR2 = \{\sum w[(F_o)^2 - (F_c)^2]^2/\sum w[(F_o)^2]^2\}^{1/2}$

Supplementary Table 3. Crystallographic data for a Cr doped UO_2 single crystal grain (SC2).

**Compound**	Cr-doped UO_2
Formula	UO_2
Formula weight (g mol^{-1})	270.03
Crystal system	Cubic
Space group	$Fm\bar{3}m$
a (Å)	5.4576(6)
b (Å)	5.4576(6)
c (Å)	5.4576(6)
α (°)	90
β (°)	90
γ (°)	90
Volume (Å^3)	162.56(5)
Z / μ (mm^{-1})	4
F(000)	432.0
d_{calcd} (g cm^{-3})	11.034
GOF	1.232
Final R_1 [a] [$I > 2\sigma(I)$]	0.0228
Final wR_2 [b] [$I > 2\sigma(I)$]	0.0477

[a] $R_1 = \sum||F_o| - |F_c||/\sum|F_o|$,

[b] $wR2 = \{\sum w[(F_o)^2 - (F_c)^2]^2/\sum w[(F_o)^2]^2\}^{1/2}$

Supplementary Information Note 4. Electron Paramagnetic Resonance (EPR) Spectroscopy

Supplementary Figure 3 provides measured EPR spectra of Cr_2O_3 measured at room temperature. The spectra become observable via the lifted antiferromagnetic coupling between Cr^{+3} cations resulting in g factor of 2.2. Notably the spectra, which is akin to a Cr^{+3}-Cr^{+3} cluster, is considerably different to that identified in the Cr-doped UO_2 single crystals (Figure 2a).

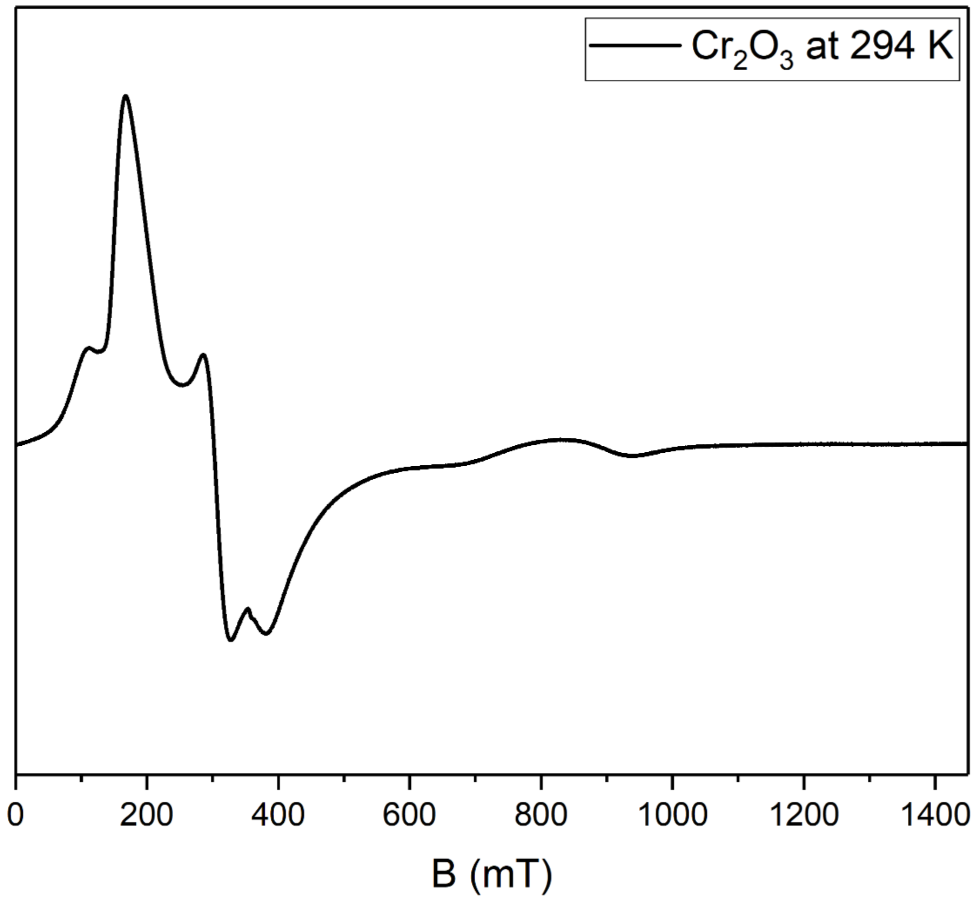

Supplementary Figure 3. EPR spectra of $Cr^{+3}{}_2O_3$ measured at room temperature.

Supplementary Information Note 5. High Energy Resolution Fluorescence Detection X-ray Absorption Near Edge Structure (HERFD-XANES) Analysis

5.1 U M_4 and L_3 Edge HERFD-XANES

HERFD-XANES measurements were performed on the U M_4-edge for the Cr-doped UO_2 powder and U L_3-edge for the Cr doped UO_2 single crystal and powder both with a UO_2 standard. The results of these are presented in Supplementary Figure 4. The results show that to limits of resolution the Cr-doped UO_2 single crystal and powder contain U^{+4} identical to that found in a UO_2 standard.

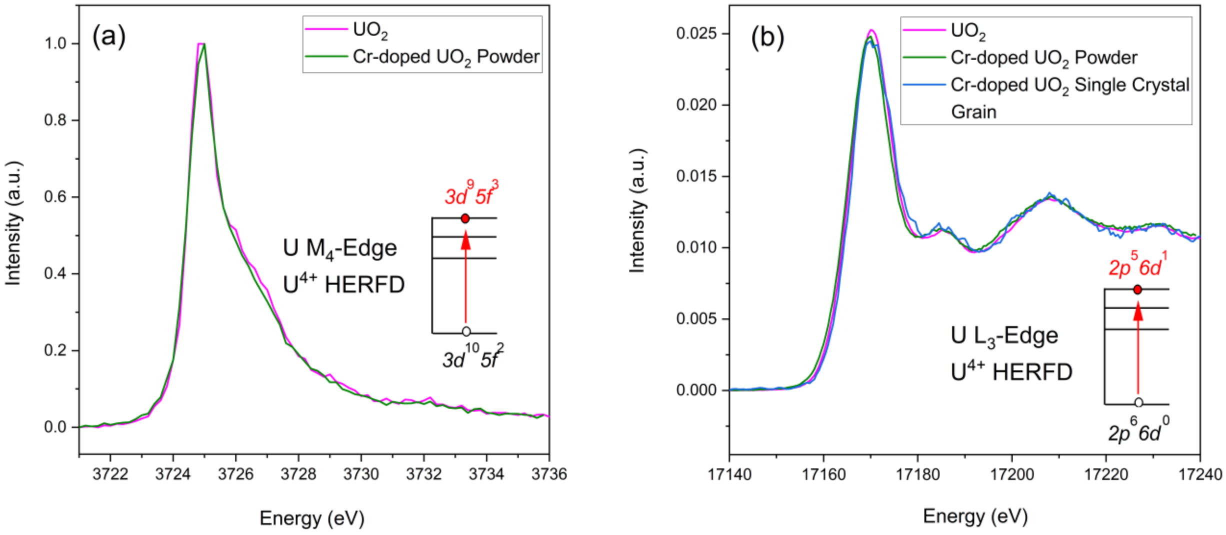

Supplementary Figure 4. (a) Normalised U M_4-edge HERFD XANES spectra for UO_2 and Cr-doped UO_2 powder with 3500 ppm addition as Cr_2O_3 and (b) Normalised U L_3-edge HERFD XANES spectra for UO_2, Cr-doped UO_2 powder with 3500 ppm addition as Cr_2O_3 and a single crystal grain. Note the consistent line shape and position of the Cr-doped UO_2 powder and single crystal grain spectra with UO_2 indicating the absence of U^{+5} and sole presence of U^{+4}. The increased noise in the single crystal grain line spectra is due to its significantly smaller size. Note a.u. denotes arbitrary units.

5.2 HERFD-XANES Iterative Transformative Factor Analysis (ITFA)

Iterative transformative factor analysis (ITFA) was used to analyse the collected HERFD-XANES spectra and quantify relative amounts of specific Cr chemical states. Supplementary Figure 5 provides the calculated ITFA components of Cr metal, Cr^{+2} (in $Cr^{+2}Cl_2$) and Cr^{+3} ($Cr^{+3}Cl_3{\cdot}6H_2O$) compared against measured Cr K-edge HERFD-XANES spectra highlighting the good reproduction of spectra from ITFA analysis.

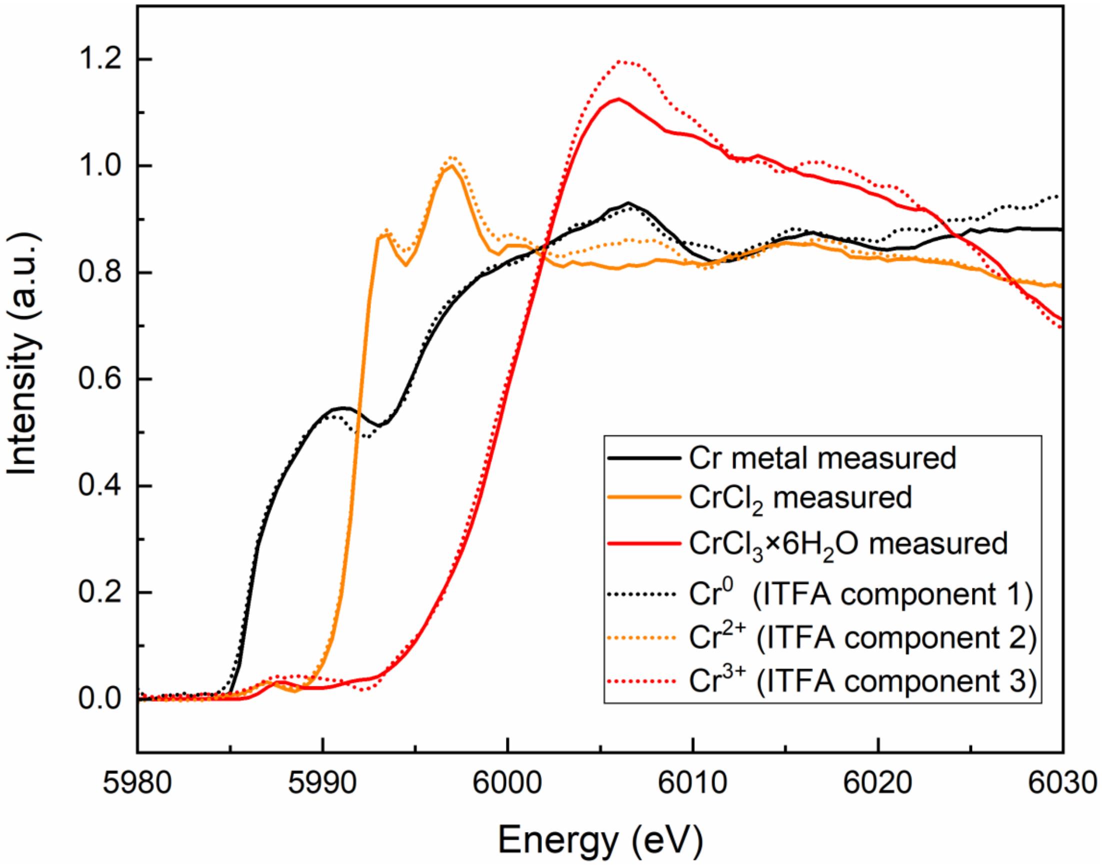

Supplementary Figure 5. Normalised Cr K-edge HERFD-XANES spectra in the range 5980 to 6030 eV for Cr for Cr^{0} (metallic), $Cr^{+2}Cl_2$ and $Cr^{+3}Cl_3{\cdot}6H_2O$ with calculated complementary ITFA components. Note a.u. denotes arbitrary units.

Supplementary Table 4. Summary of ITFA analysis for select Cr doped UO_2 single crystal grains measured using HERFD-XANES detailing Cr^0, Cr^{+2} and Cr^{+3} components. The ITFA carries a $\pm$ 5 % error.

	SC1*	SC2
Cr^0	8 %	62 %
Cr^{+2}	4 %	0 %
Cr^{+3}	88 %	48 %

*SC1 was used for presented HERFD-XANES and EXAFS analysis in main article

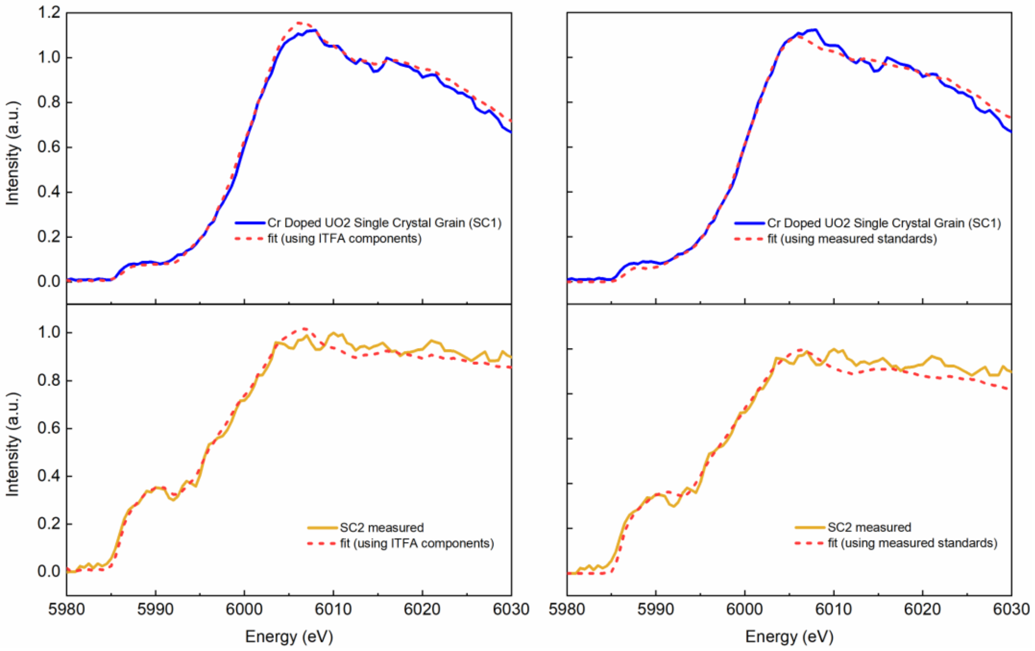

Supplementary Figure 6. Normalised Cr K-edge HERFD-XANES spectra in the range 5980 to 6030 eV for Cr doped UO_2 single crystal grains SC1 and SC2 with calculated complementary ITFA model. SC1 HERFD-XANES and EXAFS are presented in the manuscript. Note a.u. denotes arbitrary units.

Compositional analysis via ITFA carries an error of approximately $\pm$ 5 %. HERFD-XANES data were collected for several single crystal specimens. Presented in Supplementary Table 4 and Supplementary Figure 6 are SC1 (discussed in the main text, with the HERFD-XANES spectrum in Figure 2b-c), and another example crystal, SC2, originating from the same bulk material but found to contain a much higher metallic impurity content, HERFD-

XANES spectra and detailed ITFA analysis results. When the error of the ITFA analysis is considered, a trace surface impurity amount of metallic Cr, Cr^0, was found to be present in SC1 whereas a greater proportion of metallic Cr was detected in SC2. Comparing their calculated spectra from ITFA analysis to actual measured ones, the pre-edge peak at 5990 eV experiences broadening that is dependent on the amount of metallic Cr present. This effect is illustrated in Supplementary Figure 7. Accordingly, the described broadening effect is due to the presence of metallic Cr. Another notable observation from the additional Cr doped UO_2 single crystal grains is that despite present contamination with metallic Cr they appear to be near free of Cr^{+2} parasitic phases particularly when the known ITFA error is considered.

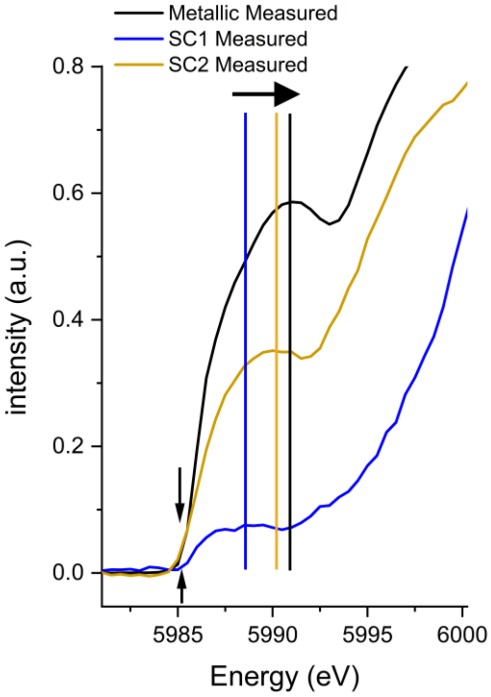

Supplementary Figure 7. Normalised Cr K-edge HERFD-XANES spectra in the range 5981 to 6001 eV for Cr doped UO_2 single crystal grains SC1 and SC2 and metallic Cr, the vertical lines and arrows are guides highlighting the broadening and drift of the pre-edge peak due to variable metallic impurity presence. Note a.u. denotes arbitrary units.

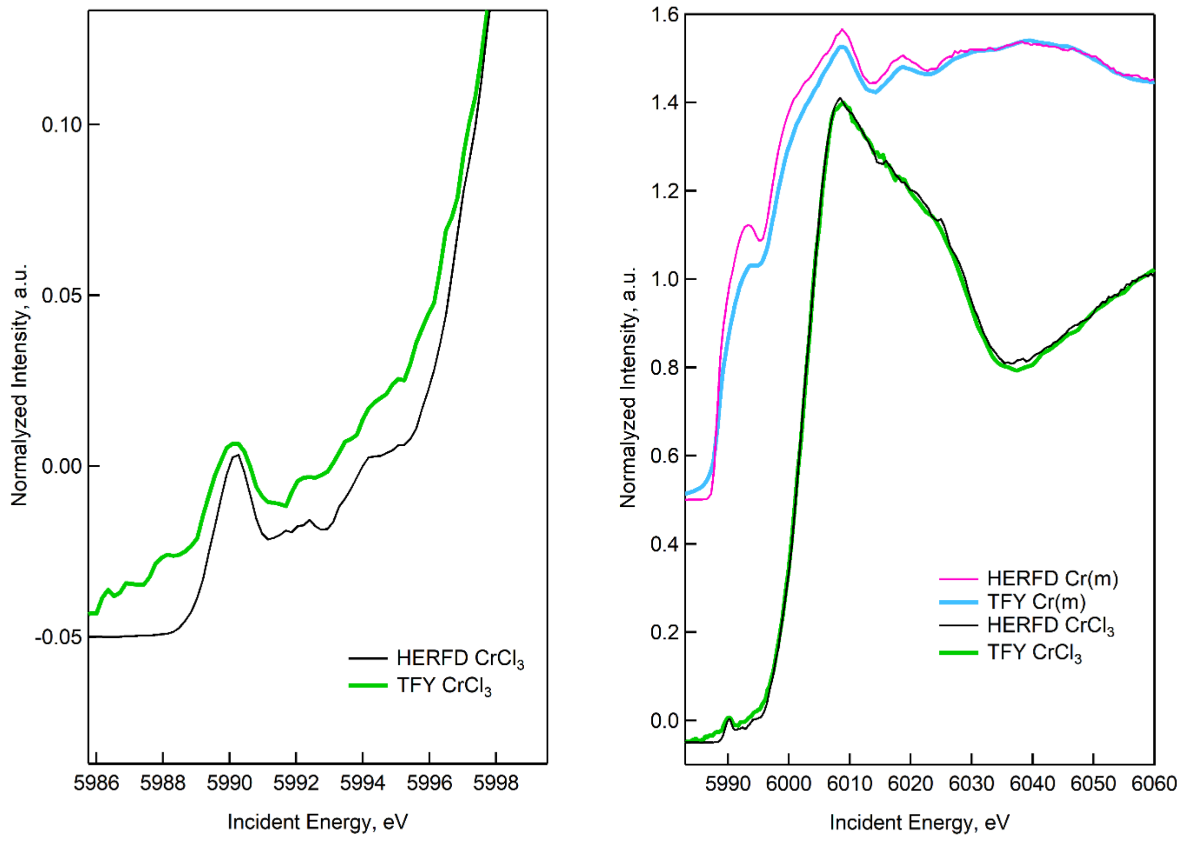

Supplementary Figure 8. Normalised XANES Cr K-edge spectra from the main- (left panel) and pre-edge (right panel) regions for $CrCl_3$ and metallic Cr taken from HERFD-XANES and from total fluorescence yield (TFY) measurements. Note a.u. denotes arbitrary units.

Supplementary Information Note 6. Ellingham Diagram

In Supplementary Figure 9 the Ellingham diagram for UO_2 is presented, where the red square marks the conditions used to synthesise Cr-doped UO_2 with 3500 ppm addition as Cr_2O_3, 1700 ^oC and μ_{O2} of -420 kJ/mol.

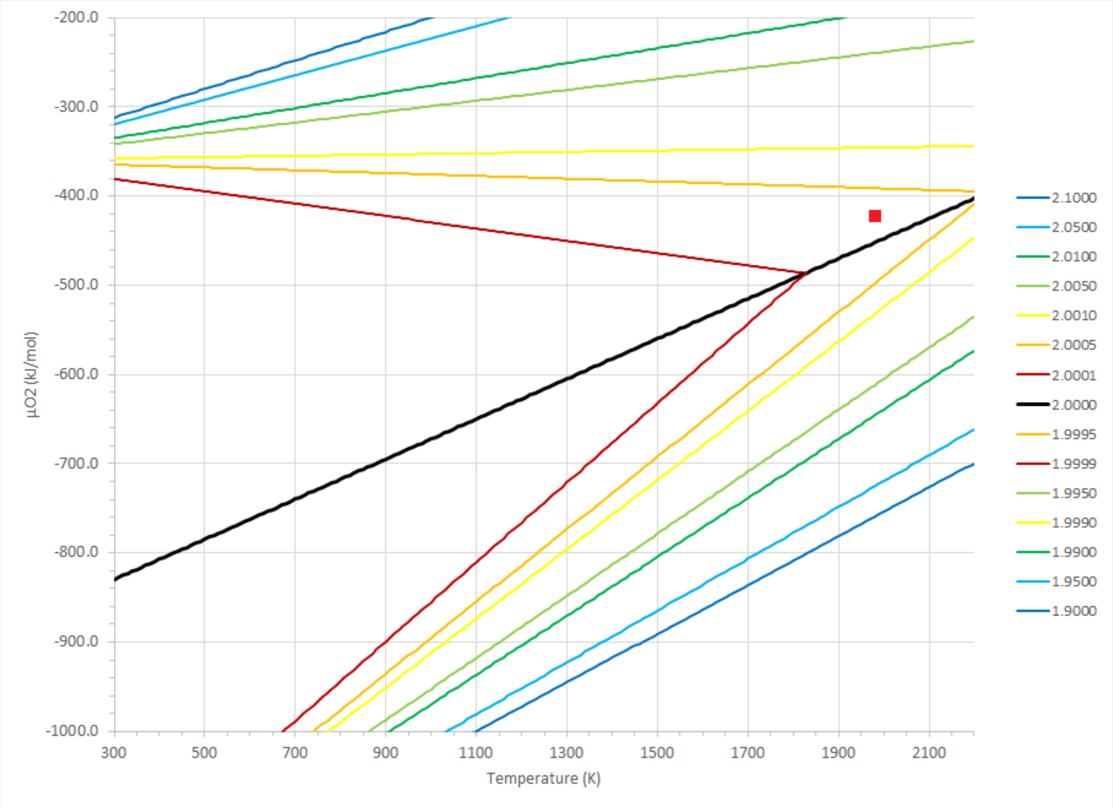

Supplementary Figure 9. The Ellingham diagram for UO_2 highlighting the conditions used to synthesise the material in the present investigation by the red square, 1973 K (1700 °C) and μ_{O2} of -420 kJ/mol.

Supplementary Information Note 7. Extended X-ray Absorption Fine Structure (EXAFS) Analysis

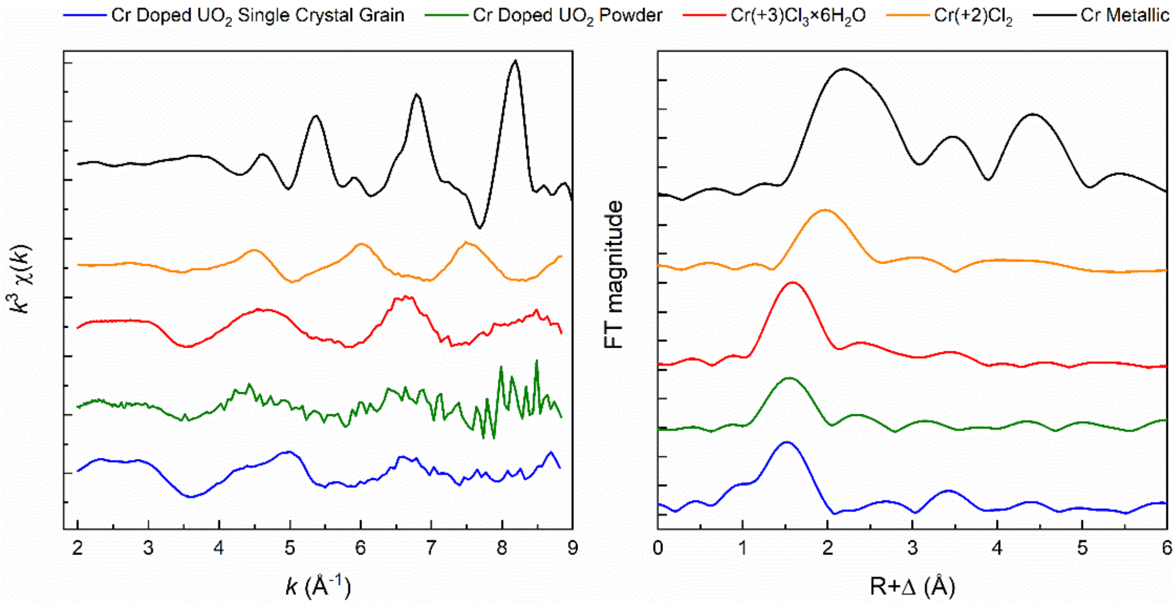

Supplementary Figure 10. k^3-weighted Cr K–edge EXAFS spectra (left) and corresponding Fourier transforms (FT) in the spectral range from 2.0 to 8.8 Å^{-1} (right) of the Cr-doped UO_2 powder, single crystal grain (SC1), and Cr standard samples. Phase shifts (Δ) are not corrected in the FTs. The single crystal specimen was measured using high energy-resolution fluorescence detection on the X-ray emission spectrometer, while powders samples were collected in fluorescence mode using an 18-element Ge-detector.

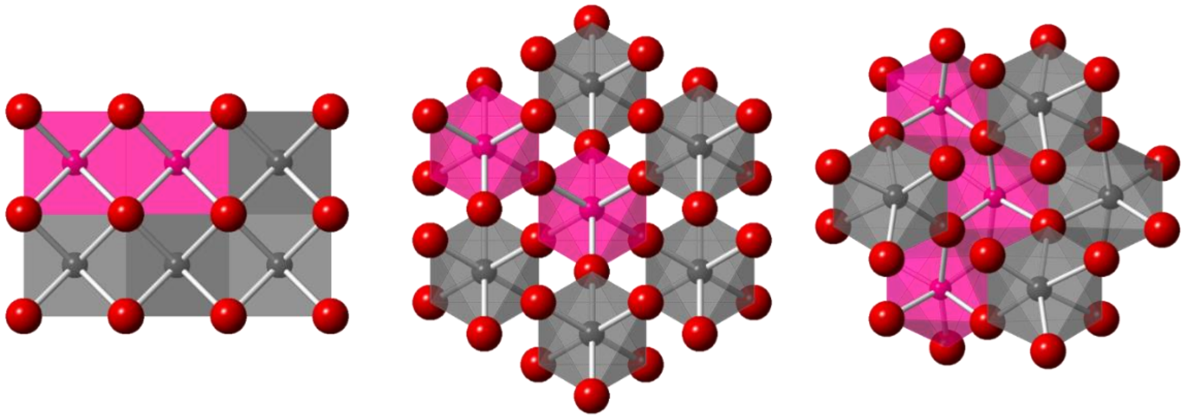

Supplementary Figure 11. FEFF models of UO_2 (left), UO_3 (middle), and $CrUO_4$ (right) used for the fitting of collected EXAFS data of the Cr-doped single crystal grain and bulk powder. Cr atoms in pink, U atoms in grey, oxygen atoms in red.
